# Back Pain in Adolescents and Young Adults with Idiopathic Scoliosis—Identifying Factors Associated with Significant Pain—A Multivariate Logistic Regression Analysis

**DOI:** 10.3390/jcm13082366

**Published:** 2024-04-18

**Authors:** Juan Bagó, Antonia Matamalas, Javier Pizones, Jesús Betegón, Judith Sánchez-Raya, Ferran Pellisé

**Affiliations:** 1 Spine Group, Vall d’Hebron Institut de Recerca (VHIR), Hospital Vall d’Hebron, 08035 Barcelona, Spain; 2 Orthopaedic Surgery, Hospital Josep Trueta, 17007 Girona, Spain; amatamalasadrover@gmail.com; 3 Orthopaedic Surgery, Hospital La Paz, 28046 Madrid, Spain; javierpizones@gmail.com; 4 Orthopaedic Surgery, Complejo Asistencial Universitario, 24008 León, Spain; jbetegonnicolas@gmail.com; 5 Physical Medicine and Rehabilitation, Hospital Vall d’Hebron, 08035 Barcelona, Spain; 34650jsr@gmail.com; 6 Spine Surgery Unit, Hospital Vall d’Hebron, 08035 Barcelona, Spain; 24361fpu@comb.cat

**Keywords:** idiopathic scoliosis, pain, psychological factors, logistic regression analysis

## Abstract

(1) **Background**: Previous data show that patients with idiopathic scoliosis (IS) can be classified into two groups according to pain intensity. This paper aims to determine which factors can independently predict the likelihood of belonging to a high-level pain group. (2) **Methods**: The study used a prospective, multicenter, cross-sectional design. Two-hundred and seventy-two patients with IS (mean age 18.1 years) (females 83.5%) were included. The sample was divided into two groups. The PAIN group comprised 101 patients (37.1%) with an average NRS of 5.3. The NO-PAIN group consisted of 171 patients (62.9%) with an average NRS of 1.1. Data on various factors such as comorbidities, family history, curve magnitude, type of treatment, absenteeism, anxiety, depression, kinesiophobia, family environment, and social relationships were collected. Statistical analysis consisted of multivariate logistic regression analysis to identify independent predictors of high-level pain. (3) **Results**: In the final model, including modifiable and non-modifiable predictors, age (OR 1.07 (1.02–1.11)); Absenteeism (OR 3.87 (1.52–9.87)), HAD anxiety (OR 1.18 (1.09–1.29)) and an indication for surgery (OR 2.87 (1.28–6.43)) were associated with an increased risk of pain. The overall model is significant at *p* = 0.0001 level and correctly predicts 72.6% of the responses. (4) **Conclusions**: Age, an indication for surgery, anxiety, and work/school absenteeism are the variables that independently determine the risk of belonging to the high-level pain group (NRS > 3).

## 1. Introduction

Currently, it appears that back pain is a significant issue for patients with idiopathic scoliosis (IS). According to recent literature reviews, the back pain prevalence among adolescents with IS exceeds 30% [1,2]. Teenagers with scoliosis are twice as likely to have back pain than those without [3,4]. Further understanding of the factors affecting pain intensity in IS patients is important because data show that pain in adolescence will determine the presence of chronic pain later on in life [5]. This fact is especially true for patients who are candidates for surgery. The preoperative pain intensity determines the pain level and perioperative opioid use, postoperative pain, and the percentage of patients with chronic pain [6,7,8,9,10].

Voepel-Lewis et al. [11] suggested the existence of two distinct clusters within the IS population based on their distinct profiles in terms of preoperative pain and somatic symptomatology. The high symptom profile group accounted for 30% of the sample and was characterized by higher depression, fatigue, pain interference, neuropathic pain, and catastrophizing.

Several authors have attempted to analyze the group differences according to pain intensity using the intuitively decided cut-off values for the numeric rating scale (NRS) [12,13,14] or the Scoliosis Research Society-22 (SRS-22) pain domain score [15]. The NRS value that identifies a patient as being in an acceptable symptomatic state was determined by Mannion et al. [16]. Using ROC analysis, they found that a threshold value of ≤3 separated patients in an acceptable state from those who were not, reaching a high predictive value (the area under the curve was 0.8). Using this methodology, Matamalas et al. [17] analyzed the differences between two cohorts of IS patients: one with a low pain level (NRS ≤ 3) and the other with a high intensity (NRS > 3). The results indicated that pain intensity was slightly influenced by age and curve size. Moreover, IS individuals who experience a significant level of pain belong to a distinct group that is defined by psychological, social, work/school, and familial factors. In addition, they reported more comorbidities and a family history of nonspecific spinal pain. This group can be distinguished from the low-level pain group.

Age, curve magnitude, and diverse psychosocial conditions (sleep disorders, depression, anxiety, or kinesiophobia) have been related to pain intensity in IS patients [12,13,14,15,18,19,20]. However, these reports were based on bivariate analysis, and the influence of some confounding variables (such as age, sex, comorbidities, or curve magnitude) on the differences between the two groups was not fully elucidated. If we consider the factors that can influence pain intensity, we can identify some that are not modifiable (such as the patient’s age, sex, or comorbidities) and others that are (such as curve magnitude, mental health, or family/work environment problems).

The objective of this paper is to determine the factors that can independently determine the likelihood of belonging to a high-level pain group through logistic regression analysis.

## 2. Materials and Methods

Study Design. This is an IRB-approved, prospective, multicenter, and cross-sectional study. Patients were recruited from the three participating centers’ outpatient scoliosis clinics. The inclusion criteria were diagnosis of IS; radiological magnitude of the major curve in the coronal plane, as measured by the Cobb angle greater than or equal to 30°; no surgical treatment; and an age between 12 and 40 years. The exclusion criteria were congenital, neuromuscular, or syndromic scoliosis. The study involved extensive interviews with parents and patients who consented to participate. The aim was to gather information on past and present comorbidities, as well as family health history, including serious diseases, scoliosis, and other spine disorders. Furthermore, information regarding age, gender, curve magnitude, and type of treatment was recorded.

Outcome Measures. The subsequent questionnaires were given to the patients for further analysis: the refined Scoliosis Research Society-22 (SRS-22r, score range 1–5) [21]; item 7 of the Core Outcome Measurement Index (COMI) [22], which assesses work/school absenteeism due to pain (score range 0–4; for multivariate analysis, the variable was categorized as No when the score = 0 and Yes, if the score was >0); and an NRS to assess self-reported pain intensity. The Hospital Anxiety and Depression Scale (HADS) [23] questionnaire was used to determine the patients’ levels of anxiety (HAD anxiety) or depression (HAD depression, score range 0–21). Additionally, the Spanish version of the Tampa Scale of Kinesiophobia (TSK) [24] (score range 11–44) and the family function APGAR [25] (score range 0–10) were also fulfilled. A set of five questions was formulated to assess the impact of the social and family environment. The patients were asked, “Do you think that any of these problems could be affecting your quality of life: the relationship with my teachers or bosses, my academic scores or my achievements at work, the relationship with my peers, lack of leisure time, and the relationship with my family?” The answer to each question was either yes or no.Statistical Analysis. SPSS version 25 software was used for the statistical analysis. Descriptive statistics included the mean, range, standard deviation, or 95% confidence interval, as required.

The categorical variables were shown as percentages. The Kolmogorov–Smirnov test was used to examine the normal distribution of continuous variables. Chi-square tests for categorical variables and Student t-test or Mann–Whitney test for continuous variables were used to compare groups.

Multivariate analysis included several binary logistic regression models. The binary dependent variable was the pain group membership (No-Pain Group NRS ≤ 3, Pain Group NRS > 3).

For Model 1, we introduced independent variables that were considered “non-modifiable”: age, sex, comorbidities, family history of scoliosis, spine pathology, or other serious diseases. For Model 2, those variables considered “modifiable” were introduced as independent variables: curve magnitude (Cobb angle), type of treatment (Observation, Brace, or Scheduled for Surgery), Absenteeism, Family APGAR, influence of the social and family environment questions, TSK, HAD anxiety, HAD depression, SRS-22 function, and SRS-22 self-image. Other variables were not included because signs of collinearity were found (VIF > 2). For Model 3, those variables from models 1 and 2 with a significance *p* ≤ 0.1 were included. An analysis using a stepwise forward introduction and a Wald chi-square to test model fit was performed for each model.

## 3. Results

From July 2018 to December 2019, 272 patients were included. The mean age was 18.1 years (range 12 to 40 years), 83.5% were female, and the average largest curve Cobb angle was 46.1° (range 30° to 96°). Regarding treatment, one hundred forty-eight patients were under Observation (no active treatment), 81 were wearing a Brace, and 43 were Scheduled for Surgery. There were 195 adolescents (age range 11 to 17 years) and 77 young adults (age range 18 to 40 years). The average pain intensity in the last month was 2.70 (SD = 2.34). The sample was divided into two groups: The PAIN group, with an NRS > 3, consisted of 101 patients (37.1%) with an average NRS of 5.3. The NO-PAIN group, with an NRS ≤ 3, consisted of 171 patients (62.9%) with an average NRS of 1.1. Table 1, Table 2 and Table 3 summarize the means (continuous variables) and the percentages (categorical variables) according to the PAIN group.

The logistic regression results using pain group membership as the categorical dependent variable were as follows:

In Model 1, variables considered “non-modifiable” were introduced as independent variables: age, sex, family history of scoliosis, spine pathology, other serious diseases, and comorbidities. The results from Model 1 (Table 4) indicate that age (OR 1.08 [95% CI 1.04–1.12]) and comorbidities (OR 1.96 [95% CI 1.03–3.73]) are significant risk factors for belonging to the PAIN group. According to the Wald chi-square statistic, the overall model is significant at the 0.0001 level. The model predicts 67.8% of the responses correctly; the Nagelkerke’s R2 is 0.13.

In Model 2, variables considered “modifiable” were introduced as independent variables: curve magnitude (Cobb angle), type of treatment (Observation, Brace, or Surgery), Absenteeism; Influence of problems in society, and familial environment, family APGAR; TSK; HAD anxiety; HAD depression; SRS22-function; and SRS22-self image. The results from Model 2 (Table 5) indicate that curve magnitude (OR 1.03 [1.00–1.05]), absenteeism (OR 3.87 [1.54–9.74]), lack of leisure time (OR 2.62 [1.30–5.26]), HAD anxiety (OR 1.13 [1.03–1.24]), and HAD depression (OR 1.15 [1.00–1.31]) determine the risk of belonging to the PAIN group. According to the Wald chi-square statistic, the overall model is significant at the 0.0001 level. The model correctly predicts 70.2% of the responses; Nagelkerke’s R2 is 0.23.

In Model 3 (Table 6), those variables from Models 1 and 2 with a significance *p* ≤ 0.1 were included: age, sex, comorbidities; family history of spinal pathology other than scoliosis; curve magnitude; type of treatment (Observation, Brace, or Surgery); absenteeism; lack of leisure time as a detriment to the quality of life; TSK; HAD anxiety; and HAD depression. The results strongly suggest that the variables independently determining the risk of belonging to the PAIN group are age (OR 1.07 [1.02–1.11)); Absenteeism (OR 3.87 [1.52–9.87]), and HAD anxiety (OR 1.18 [1.09–1.29]). The type of treatment was also significant (Wald chi-sq 8.34, *p* = 0.001). Using Observation as the reference category, Brace treatment is non-significant, but an indication for surgery was associated with the risk of pain (OR 2.87 [1.28–6.43]). The overall model is significant at a 0.0001 level (Wald Chi-sq 58.3), and Nagelkerke’s R2 is 0.27. The model correctly predicts 72.6% of the responses.

## 4. Discussion

Our findings indicate that age (OR 1.08) and comorbidities (OR 1.96) were significant non-modifiable risk factors for pain in IS patients. Among modifiable risk factors, we found that curve magnitude (OR 1.03), absenteeism (OR 3.87), lack of leisure time (OR 2.62), anxiety (OR 1.13), and depression (OR 1.15) increased the risk of pain independently. Finally, in Model 3, both modifiable and non-modifiable variables were incorporated. Age (OR 1.07), absenteeism (OR 3.87), anxiety (OR 1.18), and type of treatment (Surgery) (OR 2.87) determined the risk of PAIN group membership. The final model proved a significant goodness-of-fit and correctly identified 72.6% of the cases. This suggests that older patients with reduced physical activity due to absenteeism, anxiety disorders, and scheduled surgery were more susceptible to pain associated with IS. Other traces do not attain statistical significance, but they exhibited a distinct tendency to be associated with pain intensity (underlying medical conditions and psychological distress).

The prevalence of back pain in patients with IS increases with age, particularly after skeletal maturity [12,18,19]. Hence, it is not surprising to find that age influences pain intensity.

Absenteeism, defined as the deliberate absence from attending the place where a duty is performed, is a common complication of back pain. However, such a strong relationship found in our series of IS patients was unexpected. Subjects who reported missing work/school at least one day in the last month are 3.8 times more likely to belong to the PAIN group. Absenteeism is likely to be intertwined with fear of movement (kinesiophobia). The patients in the PAIN group had higher levels of kinesiophobia than those in the NO-PAIN group (see Table 2). However, the TSK score was not significant in the multivariate model. Fear of movement is strongly associated with pain in patients with nonspecific low-back pain [26] and has also been described in patients with IS [20,27]. This complex interplay between back pain, fear of movement, and psychosocial factors creates a vicious cycle that exacerbates absenteeism and can potentially lead to long-term disability.

Anxiety was a highly significant predictor of the risk of belonging to the high-level pain group. Numerous studies examining pain-related risk factors in patients with SI have confirmed that mental health is a significant factor. However, most of them look at mental health as a whole and do not differentiate between the anxiety and depression components [14,15,28,29]. Using the HAD questionnaire allows a separate assessment of both components. Although depression was a predictive factor in the model that includes only modifiable factors (Model 2), it was not significant in the final model (Model 3).

An indication for surgery resulted in a significant independent predictor of pain in the final model. So, patients scheduled for surgery were almost three times more likely to be in the high-level pain group. The Surgery group showed the highest pain intensity (3.3 vs. 3.0 in the Observation group and 1.7 in the Brace group). As expected, the Surgery group had the highest curve magnitude (55.9° vs. 41.2° in the Brace group and 45.8° in the Observation group). An indication for surgery is also assumed to provoke several emotions. In this scenario, anxiety may arise from uncertainty regarding the procedure, potential complications, and the outcome. However, our data showed that the indication for surgery is a predictor of pain intensity, regardless of the curve’s magnitude or the degree of anxiety experienced by the patient.

Other variables did not reach statistical significance in the final model but showed an evident tendency to be related to pain. In the final model, sex was not an independent predictor of pain. Other authors have reported similar findings [12,28,29,30]. In contrast, Voepel-Lewis et al. [11] found that girls were more likely than boys to be clustered in the high-symptom profile.

Interestingly, a family history of spinal pathology tended to be a pain predictor, whereas a family history of scoliosis did not. From the family history of spinal problems, we found that most of them involved herniated discs, neck pain, or back pain. Our results suggest that children with scoliosis whose parents have nonspecific spinal pain are more likely to have high-level pain. These findings highlight the significance of parents in influencing the symptoms and functioning of children with chronic pain [31].

We attempted to understand how conflicts in a patient’s social and family life could affect pain. Patients were asked whether their relationships with teachers/bosses, academic scores or achievements at work, relationship with peers, lack of leisure time, and their relationship with family had an impact on their quality of life. Lack of leisure time was a significant predictor in Model 2 (modifiable variables) and bordered on statistical significance in the final model. The persistent sensation of lack of leisure time, together with the impression of unfulfilled daily obligations, can generate a stress and anxiety state. The fact that this feeling of lack of time was a predictor of pain after controlling for the effect of anxiety and other factors indicates these patients have a behavioral trait, including poor time management.

The history of comorbidities did not reach statistical significance in the final model (*p* < 0.1) but was significant in Model 1, including non-modifiable variables. The most commonly reported were respiratory comorbidities (asthma and pneumonia), followed by cardiac comorbidities (congenital anomalies) and psychological comorbidities (depression, anxiety, and anorexia). The association between comorbidities and pain would not be explained by the mere presence of these comorbidities, as they were not “painful” diseases. This relationship has previously been documented in patients with low-back pain or chronic spinal pain. However, it has not been previously reported in young patients with IS.

Discussing those variables that are not significant in the final model or not included in it is also interesting. The curve magnitude was not significant in the final model, although it did reach statistical significance in Model 2 (modifiable variables). The PAIN group showed a larger Cobb angle (48.6° vs. 44.6°). Nevertheless, the difference did not exceed what is usually considered a measurement error. The literature on this subject varies remarkably, with some authors reporting an influence of the curve magnitude on pain intensity [12,18,19], while others have failed to find any difference [13,14,15]. These divergences contribute to the overall impression that the curve magnitude does not determine pain intensity.

Body image perception (SRS-22 Self-Image) also did not influence the risk of high-level pain. Our findings agree with the results of other multivariate studies [14,19,28,29], regarding the lack of relationship between pain and body image perception.

We acknowledge several study limitations. The final model correctly identified 72% of the cases, which is not a negligible number but far from ideal. We must bear in mind that one out of four patients failed to be satisfactorily classified, suggesting that other predictor variables should probably be considered and included in the model. A possible variable would be sleep quality. Wong et al. [19] observed that daytime sleepiness and insomnia were associated with episodic and/or chronic back pain in a cohort of 987 adolescent IS patients (AIS). The association between poor sleep quality and cervical and lumbar pain has also been observed in adolescents of both sexes [32,33]. Different studies have reported a significant relationship between high levels of pain catastrophizing and pain intensity in patients with AIS. Pain catastrophizing affects perioperative pain management and postoperative outcomes after AIS surgery [11,28,30]. We did not include the assessment of Pain Catastrophizing in our study. We considered that the behavioral characteristics analysis was adequately addressed by including the anxiety/depression (HAD scale), mental health (SRS-22r), and kinesiophobia (TSK) scales. Kinesiophobia and catastrophizing are both psychological factors that can significantly impact pain perception and experience. Kinesiophobia specifically focuses on the fear of movement and the potential harm therefrom. Catastrophizing has a broader focus, encompassing negative thoughts and emotions about pain itself, its consequences, and its inability to cope. Nevertheless, each can independently contribute to pain perception and disability. So, the inclusion of the variables of sleep quality and pain catastrophizing could improve the model’s accuracy.

Our study did not consider the impact of the curve type (such as Lenke’s classification) on the risk of pain. It could be argued that, in patients with lumbar curves, pain could be related to the existence of disc and/or facet degeneration processes. However, considering the mean age of our cohort, the presence of significant degenerative phenomena does not appear likely. Moreover, several patients had received other exams that were not gathered for the present analysis. In particular, patients with high pain were specifically evaluated (bone scans, MRI) to rule out other causes of pain apart from scoliosis. At this point, we must realize that we cannot explain the origin of pain based on organic findings related to scoliosis. Therefore, it is imperative to conduct additional research to gain a more in-depth understanding of the nature of pain.

Our results are in the same direction as those reported by Voepel-Lewis et al. [9,11]: within the AIS patient population, a cluster with a “behavioral pain vulnerable profile” can be identified. This group would include approximately 30% of the patients. In our setting, this cluster would include patients with pain intensity > 3, scheduled surgery, anxiety traces, and reported work or school absenteeism. Some additional traits observed in these patients would include a family history of nonspecific neck/lumbar pain, past/current comorbidities, and conflicts in a patient’s social and family life, manifested in a persistent sensation of lack of leisure time. Interestingly, a similar cluster was observed by Pellisé et al. [34] in a cohort of 1470 adolescents (mean age 15 y.o.) in which 22.3% of respondents reported back pain not limited to the lumbar region and a mean intensity of 6.

From a daily clinical practice perspective, we suggest that AIS patients who report a significant level of pain should initially undergo an investigation to rule out an organic cause of the pain. Second, the psychological, social, and family profiles should be explored. If the patient is deemed to exhibit a significant pain and symptom profile, it is imperative to establish a red flag, particularly in the event of undergoing surgery. The patient must receive tailored treatment and specialized support as this profile will predict a high-level pain pathway. Providing clear and detailed information about the surgical procedure and its possible outcomes is imperative to reduce patient anxiety. Managing anxiety seems to be the crucial point in reducing the pain level. To effectively reduce absenteeism, it is necessary to address the complex interplay between back pain, fear of movement, and psychosocial factors. Cognitive-behavioral therapy has shown promising results in reducing pain, disability, and kinesiophobia in adults with IS [35]. New research underway will test the efficacy of internet-delivered psychosocial intervention to reduce postoperative pain in adolescents undergoing spinal fusion [36].

## 5. Conclusions

It is possible to identify a high-level pain group (NRS > 3) comprising approximately 30% of the sample of AIS patients. The variables that independently determine the risk of belonging to this group were age, an indication for surgery, anxiety, and work/school absenteeism. Other variables were not statistically significant, but they showed a distinct tendency to be associated with pain intensity (underlying medical conditions and psychological distress). The degree of pain was not affected by variables that affected the scoliosis description, such as the curve magnitude or body image. Nevertheless, this model misclassifies one in four patients and requires further improvement. This would probably require the inclusion of additional predictors in the model, such as pain catastrophizing or sleep quality. An early and accurate identification of this group of patients would allow for specific management to modify the pain itinerary, particularly in patients who are candidates for surgical treatment, and facilitate targeted treatments to reduce long-term negative postoperative outcomes.

## Figures and Tables

**Table 1:** Mean/percentage for each variable and pain group. Non-modifiable variables.

	NO-PAIN	PAIN	*p*
Age (y.o.)	16.5	20.9	0.0001
Sex (% Females)	80.1	89.1	0.06
Family history of scoliosis (%)	33.5	41.6	0.1
Other spine diseases in the family (%)	13.6	24	0.04
Other severe diseases in the family (%)	20	18.2	0.7
Comorbidities (%)	14.7	33.7	0.0001

**Table 2:** Mean/percentage for each variable and pain group. Modifiable variables.

	NO-PAIN	PAIN	*p*
Cobb (°)	44.6	48.6	0.025
SRS-22 function	3.83	3.64	0.1
SRS-22 image	3.23	3.11	0.3
TSK	21.7	23.6	0.023
HADS anxiety	4.9	7.2	0.0001
HADS depression	1.9	3.3	0.0001
Family APGAR	8.8	8.58	0.3
Absenteeism (% patients with COMI#7 > 0)	4.7	23.8	0.0001

**Table 3:** Percentage of patients reporting problems in the social and family environment.

	NO-PAIN	PAIN	*p*
Relationship with teachers/bosses	2.40%	11%	0.005
Academic/work success	13.60%	31%	0.001
Relationship with peers	7.70%	17%	0.02
Lack of leisure time	10.70%	30%	0.0001
Family relationships	4%	16%	0.001

**Table 4:** Binary logistic regression. Model 1. Dependent variable: Pain Group.

	B	Wald	OR (CI 95%)	*p*
Age	0.08	14.8	1.08 (1.04–1.12)	0.0001
Sex				0.1
Comorbidities	0.67	4.22	1.96 (1.03–3.73)	0.02
Family history of scoliosis				0.4
Family history of spine pathology other than scoliosis				0.09
Family history of other serious diseases				0.6

**Table 5:** Binary logistic regression. Model 2. Dependent variable: Pain Group.

	B	Wald	OR (CI 95%)	*p*
Cobb angle	0.03	7.96	1.03 (1.00–1.05)	0.005
TSK				0.1
HAD anxiety	0.13	7.66	1.13 (1.03–1.24)	0.006
Had depression	0.14	4.35	1.15 (1.00–1.31)	0.03
Type of treatment				0.07
Absenteeism	1.35	8.3	3.87 (1.54–9.74)	0.04
SRS-22 function				0.4
SRS-22 image				0.9
Family APGAR				0.9
relationship with teachers or bosses				0.4
academic scores or achievements at work				0.3
relationship with peers				0.2
lack of leisure time	0.96	7.36	2.62 (1.30–5.26)	0.007

**Table 6:** Binary logistic regression. Model 3. Dependent variable: Pain Group.

	B	Wald	OR (CI 95%)	*p*
Cobb angle				0.3
TSK				0.5
HAD anxiety	0.17	16.2	1.18 (1.09–1.29)	0.0001
HAD depression				0.15
Type of treatment Observation (ref) Brace Surgery	−0.11 1.05	0.09 6.57	0.89 (0.43–1.85) 2.87 (1.28–6.43)	0.01 0.76 0.01
Absenteeism	1.35	8.05	3.87 (1.52–9.87)	0.005
SRS-22 function				0.4
SRS-22 image				0.9
Age	0.06	8.27	1.07 (1.02–1.11)	0.004
Sex				0.06
Comorbidities				0.1
lack of leisure time				0.055

## Data Availability

The datasets presented in this article are not readily available because they are part of an ongoing study.
